# Bacterial Metal Resistance: Coping with Copper without Cooperativity?

**DOI:** 10.1128/mBio.00653-21

**Published:** 2021-06-15

**Authors:** Nicholas P. Greene, Vassilis Koronakis

**Affiliations:** a Department of Pathology, University of Cambridge, Cambridge, United Kingdom

**Keywords:** tripartite efflux pumps, bacterial metal resistance, membrane proteins

## Abstract

In Escherichia coli and other Gram-negative bacteria, tripartite efflux pumps (TEPs) span the entire cell envelope and serve to remove noxious molecules from the cell. CusBCA is a TEP responsible for copper and silver detoxification in E. coli powered by the resistance-nodulation-cell division (RND) transporter, CusA. In a recent study, Moseng et al. (M. A. Moseng, M. Lyu, T. Pipatpolkai, P. Glaza, et al., mBio 12:e00452-21, 2021, https://dx.doi.org/10.1128/mBio.00452-21) obtained cryo-electron microscopy (cryo-EM) structures of CusA trimers in the presence of copper. The multiple conformations revealed suggest that the three monomers function independently within the CusA trimer, contrary to the cooperative mechanism proposed for the multidrug exporting RND transporter, AcrB. The work prompts consideration of the mechanism of this class of transporter and provides a basis to underpin further studies of TEPs important for bacterial survival.

## COMMENTARY

Copper is an essential micronutrient for life with its ability to cycle between two oxidation states exploited in respiratory electron transport chains and the neutralization of reactive oxygen species by superoxide dismutases. However, this reactivity is a double-edged sword, as unchecked, redox cycling leads to oxidative stress and excess copper can displace other essential metal cofactors, resulting in protein dysfunction. This toxicity is exploited by macrophages and other immune cells in the innate immune system as an antibacterial weapon (1). Bacteria have therefore evolved mechanisms to sense and neutralize the deleterious effect of free copper (2), a common strategy being to extrude excess copper from the cell using membrane transporters (3). In Gram-negative bacteria, tripartite efflux pumps (TEPs) are multicomponent assemblies that extend across the cell envelope and act to detoxify noxious molecules. Commonly, these pumps can efflux a range of structurally diverse compounds, including multiple antibiotics, but a subset of TEPs are dedicated to the removal of specific metal ions (4). One such example, Escherichia coli CusCBA, comprises an inner membrane transporter component, CusA, linked to a trimeric outer membrane channel, CusC, by a hexameric ring of the periplasmic adaptor protein CusB. The engine of this molecular machine is CusA, a resistance-nodulation-cell division (RND) transporter that utilizes the power of the transmembrane proton electrochemical gradient to effect efflux of copper and silver ions through the CusC tunnel and out of the cell. Recent work by Moseng and colleagues (5) provides further insight into how this is achieved.

Structures of trimeric CusA have been solved by X-ray crystallography and reveal 12 transmembrane helices and an extensive periplasmic domain made up of regions between TM1/2 and TM7/8. The metal binding site within a periplasmic cleft includes methionine residues that can specifically coordinate the copper ion (6). The overall fold is similar to RND transporters such as AcrB and MexB that form TEPs responsible for the expulsion of bile salts and antibiotics from E. coli and Pseudomonas aeruginosa. Multiple structures of AcrB have been obtained, including drug-bound states in which each monomer adopts a different conformation allowing access, binding, and extrusion of the drug, respectively. These observations led to the proposal of a functionally rotating mechanism in which monomers cycle between each state in a cooperative fashion (7, 8).

The RND subfamily responsible for efflux of metal ions has been less extensively studied, and there are key differences between the heavy metal pumps, including CusA, and the drug efflux RND class. The latter have multiple binding sites, entry paths, and a broad substrate specificity (9). Conversely, metal efflux pumps are specific, sometimes for a single oxidation state of a metal ion, and lack specific structural features important for drug efflux. In a recent study, Moseng and colleagues (5) utilize a combination of cryo-electron microscopy (cryo-EM) to obtain multiple CusA structures in different states from a single sample. By solving the structures of CusA protein reconstituted in E. coli lipids, concerns that detergent could artifactually affect the structure are ameliorated. Furthermore, the use of cryo-EM precludes the conformation of individual protomers being influenced by the crystal contacts between trimers that occur in X-ray crystallographic studies. Two states of CusA are revealed, a copper-free “extrusion state” and a binding state in which conformational changes in the periplasmic domain open a cleft in which the copper binding site is situated. A single Cu(I) ion is bound, not just by methionines as originally proposed, but also by a glutamate consistent with X-ray absorption studies indicating that an oxygen or nitrogen atom was involved in copper binding (10). Crucially, within the same sample, the authors observed symmetric empty and fully copper-bound states as well as asymmetric states in which one or two of the monomers contained copper. The coexistence of these structures within a single sample indicates an absence of cooperativity between individual monomers, suggesting they could operate independently during efflux, in contrast to the cooperative mechanism of AcrB.

An important point is that the CusA structures were solved in the absence of the other pump components. CusA forms multiple contacts with the hexameric funnel formed by the periplasmic adaptor protein CusB within the assembled pump (11). CusB does not simply provide a passive link between the other two pump components but can itself bind copper, possibly to regulate pump assembly (12), but the N-terminal region responsible for this activity is not resolved in the CusB crystal structure (11). Furthermore, an accessory copper binding, periplasmic factor, CusF is required for resistance and may function by transferring copper to CusB (13) or directly to CusA from the periplasm (10). CusF has no equivalent in the drug efflux pumps, and the precise site and mechanism of interaction are not clear. The extent to which contacts with these other proteins can influence the state of individual CusA monomers, and how this would affect the activity of the pump as a functional trimer therefore remains to be seen. Notably, a small, previously uncharacterized membrane protein, AcrZ, regulates the activity of AcrB (14, 15), so it is possible that as yet unknown factors could influence the activity of the CusBCA pump *in vivo*.

These caveats aside, could the results observed reflect genuine differences between the metal- and drug-exporting RND transporters? Interestingly, the same group has reported crystal structures and *in vitro* fluorescence data that suggested a drug-exporting RND pump from Campylobacter jejuni could also operate noncooperatively (16). If this is reflective of *in vivo* function, then the trimeric state would presumably reflect a stable state, rather than a requirement for function, since trimeric transporters are not a requisite for TEP complex formation (17, 18). Clearly, further studies are needed to resolve the wealth of data supporting AcrB cooperative function with these newer observations, and how the different states observed reflect the subtleties of pump mechanism.

CusBCA has the capability of removing copper from both periplasmic and cytoplasmic compartments. The CusA structures reveal a copper permeation route along a network of methionines within the transmembrane domain and demonstrate how these could be coupled with proton transport to open and close this cytoplasmic copper pathway. Genetic evidence suggests that an inner membrane ATPase, CopA, may be the primary route of copper extrusion from the cytoplasm in which case the transmembrane copper pathway delineated here may therefore act as a cytoplasmic sensor, rather than a major efflux pathway (19), with copper extrusion occurring predominantly through the periplasmic route. In common with other efflux pumps, though more than one efflux pathway exists, CusA may act primarily as a periplasmic “vacuum cleaner” for copper and silver ions (20).

To more completely understand the mechanism of the pump, the structure of assembled CusBCA or another metal effluxing TEP in different states would be of obvious benefit. Structures of entire drug effuxing TEPs have been solved by cryo-EM, but this is not a trivial undertaking and high-resolution structures have often relied on stabilizing cross-links/fusions to maintain the stability of the entire complex or reconstitution from individually purified components (15, 21, 22). Nevertheless, in combination with biophysical techniques, the power of cryo-EM to obtain information from a heterogeneous sample has the potential to resolve the apparent differences in cooperativity reported for RND transporters. The cooperative state of AcrB and MexB are evident in the drug-bound assembled tripartite pump (21–23), and it would be telling to observe the state of CusA in the fully assembled apparatus. Moseng and colleagues used molecular dynamics simulations to understand the transition between states of the CusA pump. As computational power increases and more structural information evolves, such efforts have the potential to reveal transient states and animate transitions between static structural snapshots to capture the molecular details of efflux.

Such insights into the metal efflux mechanism are more than a matter of academic interest. CusBCA-type TEPs are absent from eukaryotes but important for the survival of Gram-negative pathogens and therefore constitute potential targets for antimicrobial therapy. Attempts have already been made to interfere with the function of the CusBCA pump using small molecule inhibition (24), and further studies have the potential to reveal new avenues to exploit.
